# Academic and Socio-Emotional Experiences of a Twice-Exceptional Student

**DOI:** 10.3390/bs15101349

**Published:** 2025-10-02

**Authors:** Davut Açar, Muhammet Davut Gül

**Affiliations:** 1 Department of Child Development, Vocational School of Health Services, Hakkari University, Hakkari 30000, Türkiye; davutacar@hakkari.edu.tr; 2 Department of Special Education, Faculty of Education, Tasliciftlik Campus, Tokat Gaziosmanpasa University, Tokat 60150, Türkiye

**Keywords:** academic, socio-emotional, twice-exceptional

## Abstract

Twice-exceptional students, who are both gifted and present with characteristics of neurodiversity such as Autism Spectrum Disorder (ASD), possess distinctive academic and socio-emotional needs that necessitate individualized educational strategies. This qualitative case study explores the academic and socio-emotional experiences of Murat, an eighth-grade learner identified as gifted and diagnosed with ASD, from the perspectives of the student himself, his mother, and his teachers. Data were collected through semi-structured interviews and analyzed using Braun and Clarke’s six-phase reflexive thematic analysis. The findings revealed that Murat achieved success in mathematics and science, particularly within enriched, strength-oriented environments that accommodated his sensory sensitivities. Despite challenges in social skills and group participation, he benefited considerably from teacher scaffolding and interactive pedagogies. His mother’s active engagement and strong family–school collaboration emerged as pivotal factors in his developmental progress. This study extends beyond individual challenges to highlight the potential strengths that arise from by the intersection of neurodiversity and giftedness. Additionally, it contributes to the limited body of literature exploring how the notion of twice-exceptionality manifests within underrepresented educational contexts. Future research could investigate diverse socio-cultural contexts and develop strategies to enhance teacher preparation and family engagement in supporting gifted learners with ASD.

## 1. Introduction

Individual differences in cognitive processes and learning styles contribute to the diverse educational trajectories of students. Among these differences, some students demonstrate giftedness while simultaneously presenting with neurodevelopmental traits, leading to their categorization as twice-exceptional (2e) (Reis et al., 2014). This designation encompasses individuals who exhibit high intellectual potential while also presenting with conditions such as Attention Deficit Hyperactivity Disorder (ADHD), Autism Spectrum Disorder (ASD), or Learning Disabilities (LD) (Foley Nicpon et al., 2011). Supporting 2e students poses a dual challenge, requiring the concurrent cultivation of their strengths alongside the provision of targeted support for their areas of need (Foley-Nicpon & Assouline, 2020). Importantly, both giftedness and neurodevelopmental diversity bring not only strengths or deficits but also cognitive, social, and emotional complexities. For instance, giftedness may be associated with perfectionism, overexcitability, or anxiety, whereas neurodiversity may afford distinctive problem-solving strategies, creativity, and intense focus (Baum et al., 2014; Neihart, 2007).

To effectively support 2e students, educational strategies must acknowledge this complexity and create environments that simultaneously address challenges and capitalize on strengths (Reis et al., 2014). Studies focusing on 2e students with ASD have identified distinct patterns of high academic performance in specific domains; however, these strengths are frequently accompanied by psychosocial challenges such as anxiety, depression, and social withdrawal (Rubenstein et al., 2015). Prior studies have examined parental perspectives (Rubenstein et al., 2015), gender differences (Assouline et al., 2009), academic profiles (Assouline et al., 2012; Cain et al., 2019; Reis et al., 2022a; Wu et al., 2019), psychosocial functioning (Burger-Veltmeijer et al., 2016; Doobay et al., 2014; Foley Nicpon et al., 2010), and language development (Melogno et al., 2015). However, limited research exists, particularly within the Turkish educational context, that explores the experiences of 2e students with ASD from multiple perspectives. In Turkey, research on twice-exceptional (2e) students is limited in both theoretical and practical (empirical) areas. Much of the existing work is theoretical or descriptive, including conceptual overviews and analyses of graduate theses (Bildiren & Fırat, 2020; Sağlam & Çiftçi, 2022; B. Arslan, 2024; Yılmaz-Yenioğlu & Melekoğlu, 2021). A few studies have examined educators’ views using metaphorical or interpretive methods (Sakar & Köksal, 2021; Fırat & Bildiren, 2023). Few studies directly engage 2e learners with participatory methods (Yenioğlu et al., 2022; Bildiren et al., 2023; Sıkıcıkoğlu et al., 2024). This gap highlights the need for more practical, well-designed studies that document the lived experiences of twice-exceptional students. This study seeks to address this gap by examining the academic and socio-emotional experiences of a gifted learner with ASD through the perspectives of the student, his mother, and his teachers.

### 1.1. Academic Skills of 2e Students

For 2e learners, academic achievement often stems from strong intrinsic motivation and sustained interest in particular content domains. Cain et al. (2019), for example, found that 2e learners consistently outperformed both their neurotypical and neurodiverse peers over time. Engagement in enrichment activities grounded in students’ interests fosters both academic development and social confidence. Ensuring access to enriched learning opportunities and strengths-oriented instruction is therefore essential for this population (Reis et al., 2022b). Nonetheless, such gains can be compromised by the social-emotional challenges commonly associated with ASD (Foley Nicpon et al., 2010). Tailored interventions from educators, psychologists, and therapists are critical to promote holistic development (Baum et al., 2014). A persistent challenge, however, is the limited awareness and insufficient training among teachers in recognizing and addressing the needs of 2e learners (Alsamani et al., 2023). As Rubenstein et al. (2015) observed, many parents struggle to locate educational settings that simultaneously accommodate both giftedness and disability, frequently assuming advocacy and resource-seeking roles themselves. This issue tends to be more pronounced in resource-limited or rural contexts. Ultimately, a combination of individualized academic programming aligned with learners’ interests and active extracurricular engagement is vital to the overall well-being and success of 2e learners (Foley Nicpon et al., 2011; Reis et al., 2022a).

### 1.2. Socio-Emotional Skills of 2e Students

Burger-Veltmeijer et al. (2016) highlighted that 2e students often display inconsistent cognitive and adaptive profiles. While they may demonstrate strong performance in abstract reasoning and tasks requiring logical problem-solving, difficulties with executive functioning, reciprocal social interaction, and everyday self-care skills are common. Intense special interests are often misperceived by peers or educators, and developmental asynchrony can further complicate social integration. Socio-emotional and behavioral immaturity is also frequently observed, particularly in relation to implicit social norms and developmentally appropriate behaviors (Foley Nicpon et al., 2010). This disconnect between cognitive strengths and socio-emotional challenges may contribute to heightened anxiety, diminished self-concept, and symptoms of depression (Wu et al., 2019; Doobay et al., 2014).

What renders diagnosis and intervention even more complex is the presence of overlapping characteristics associated with both giftedness and ASD, such as sensory sensitivities, hyperfocus, and heightened intellectual curiosity (Neihart, 2007). Addressing these needs necessitates a multifaceted approach that includes peer-awareness programs, social skills interventions, and family support systems. Beljan et al. (2006) underscored the importance of fostering inclusive environments that acknowledge and nurture both the abilities and vulnerabilities of 2e learners. Families of 2e learners frequently experience significant emotional and financial strains. Counseling and parent education programs can equip caregivers with strategies to more effectively support their children’s twice-exceptional needs (Reis et al., 2014). In light of this complexity, the present study adopts a holistic framework and seeks to provide an in-depth examination of both the academic and socio-emotional experiences of a gifted learner with ASD within the Turkish educational context. Drawing on in-depth, semi-structured interviews with the student, his mother, and his teachers, the study integrates multiple perspectives to analyze the challenges encountered as well as the mechanisms of effective support.

To achieve a more systematic comprehension of the multifaceted dimensions of twice-exceptionality and its contextual interdependencies, the present investigation employs Bronfenbrenner’s ecological systems theory as its conceptual framework. This paradigm posits human development as an emergent process influenced by reciprocal interactions between the individual and the stratified environmental systems surrounding them (Bronfenbrenner, 1979). The empirical data collected in this study were interpreted through the prism of selected strata within the ecological model, with particular emphasis on the microsystem—encompassing proximal settings such as the familial and scholastic milieus—and the mesosystem, which delineates the interconnections among these domains. Employing this theoretical lens, the inquiry endeavors to comprehensively examine not merely the participant’s intrinsic aptitudes and impediments, but also the exogenous variables that modulate their encounters. Such factors encompass pedagogical architectures, educator dispositions, and familial interrelations, all of which collectively inform the experiential trajectories of a gifted student with autism spectrum disorder in the Turkish educational landscape.

## 2. Materials and Methods

This study employed a qualitative case study design. A case study refers to the examination of phenomena experienced by individuals, groups, communities, or units within a defined research framework (Creswell & Poth, 2016). The design facilitates an in-depth exploration of phenomena and their holistic understanding within real-world contexts (McMillan, 2012). Single-case designs are often selected for purposes such as the corroboration of theoretical insights, the documentation of rare or atypical cases, longitudinal tracking of similar phenomena, and the exploration of hard-to-access contexts (Yin, 2003). Within this framework, the data collection strategy comprised six semi-structured interviews: two with the student, two with the mother, and two with the teachers. The collected data were analyzed using Braun and Clarke’s (2021) reflexive thematic analysis. The details of this analytic approach are elaborated in subsequent sections.

### 2.1. Participants

The study participants consisted of Murat, an 8th-grade student identified as gifted and diagnosed with ASD, his mother, and two teachers employed at Science and Art Centers (SACs) (See Table 1). SACs are state-funded, part-time centers that provide supplementary educational programs for gifted students at the elementary, middle, and high school levels, with a strong emphasis on enrichment and ability grouping (Sak, 2012). Student admission involves teacher nomination, group intelligence testing, individual intelligence assessment, and committee-based evaluation across talent domains such as intellectual ability, visual arts, and music. The curriculum follows a five-stage model—orientation, support, talent identification, talent development, and engagement in project work—highlighting project- and problem-based learning (Güçyeter et al., 2017).

Murat, who presented with an unremarkable birth history, is enrolled in a public school and, due to his giftedness, also attends the state-funded SACs. His giftedness was formally identified through the Anadolu-Sak Intelligence Scale (ASIS) at the onset of his enrollment in SACs. ASIS, an individually administered cognitive ability test standardized in Turkey in 2016 for Turkish-speaking children aged 4 to 12, is grounded in the Cattell–Horn–Carroll model of intelligence and comprises 256 items across seven subtests and three factors (Sözel et al., 2018). Its psychometric reliability and validity have been established through exploratory and confirmatory factor analyses (D. Arslan & Sak, 2023; Tamul et al., 2020). Murat received a diagnosis of ASD at the age of eight. Despite demonstrating high academic potential in mathematics and science, he faces challenges related to social reciprocity and behavioral adaptation. These challenges necessitate tailored strategies within his educational process and the adoption of individualized instructional approaches. At the time of data collection, Murat was preparing for the High School Transition System (HSTS), a centralized system implemented in Turkey since 2018 to evaluate students’ ability to transfer acquired knowledge into applied skills (Ar et al., 2023). The exam assesses core subjects including Turkish, mathematics, science, social studies, and religious culture. Murat aspired to pursue a high-quality secondary education and eventually gain admission to medical school. According to the latest updates during the research process, he ranked within the top 3% of students nationwide in the HSTS and was admitted to a Science High School.

A pilot testing process was conducted with another student who presented comparable characteristics to those associated with both ASD and giftedness. This stage was used to assess the comprehensibility and appropriateness of the interview questions, leading to minor linguistic refinements. The interview protocol was developed with the expert input of two academics specializing in special education and gifted education. The participating student was fully informed about the study, and both parental informed consent and the student’s verbal assent were obtained.

### 2.2. Data Collection and Procedure

In line with qualitative research methodology, the study employed a semi-structured interview protocol consisting of open-ended questions (Creswell & Poth, 2016). The interview items were intended to provide an in-depth exploration of the academic and socio-emotional experiences of twice-exceptional individuals. During the literature review, themes were extracted primarily from works such as Kim (2016) and Foley-Nicpon (2021), which informed the structuring of the interview questions. The draft protocol was reviewed by two academic consultants with expertise in special education and gifted education. These experts provided input regarding content validity and suitability for the participant population and are formally acknowledged in the acknowledgments section. Based on their feedback, a pilot study was conducted with a student diagnosed with both ASD and giftedness who presented comparable characteristics. The pilot study examined the clarity and comprehensibility of the questions and allowed for the identification and elimination of potentially leading or biased wording. As a result of the pilot study, several items were linguistically refined and reorganized to facilitate thoughtful engagement in participants’ responses.

The data collection process was conducted in May of the 2024–2025 academic year. Once the participating student was identified, the family and teachers were contacted, the aims of the study were explained, and written parental consent was obtained. The student was also provided with detailed information about the process, and in addition to written consent, the student’s “voluntary assent” was secured. All interviews were conducted by the first author of the study in a quiet setting at the center. In total, six interviews were carried out: two with the student, two with the mother, and two with the teachers. Each interview lasted approximately 25–30 min, was audio-recorded with prior permission, and transcribed verbatim. In accordance with confidentiality principles, all names were replaced with pseudonyms and identifying details were removed from the transcripts.

### 2.3. Data Analysis

The collected data were analyzed using the reflexive thematic analysis method developed by Braun and Clarke (2021). This approach is a qualitative method that acknowledges the researcher’s subjective position and aims to provide a critical and flexible lens for engaging with the data. The process followed a six-phase structure: familiarization with the data, generation of initial codes, development of themes, review of themes, definition of themes, and reporting. First, two researchers (coder 1 and coder 2) repeatedly read four different interview transcripts to gain familiarity with the data. Subsequently, the first author (coder 1) generated open codes that captured significant meaning units. Throughout the coding process, mnemonic memos were kept in order to identify patterns across the data and establish conceptual connections (Charmaz, 2006). This iterative procedure ensured the evaluation of codes in terms of both consistency and contextual depth.

Subsequently, the codes were compared by the two researchers and consolidated through consensus. Three main themes were identified for the student, four for the mother, and three for the teachers. The alignment of the themes with both the codes and the overall data set was carefully examined, after which their labels were revised and their boundaries refined. Finally, vivid and illustrative participant quotations were selected to represent each theme and were reported in relation to the research questions and the existing literature.

### 2.4. Validity

To enhance the validity of the findings and the trustworthiness of interpretations based on qualitative data, this study employed several strategies commonly adopted in qualitative research. These included member checking, expert review, and the provision of thick, rich descriptions (Patton, 2014). As part of the member checking process, the analyzed findings and themes were shared with the participants, who confirmed the accuracy of the statements and the contextual integrity. During the expert review stage, specialists from two different academic fields (gifted education and special education) provided feedback on the analytic process and evaluated the alignment of the themes with the research questions.

Consistent with Braun and Clarke’s (2021) reflexive thematic analysis approach, the analytic process was conducted with explicit consideration of the researcher’s subjective position. During coding and theme development, analytic reflexivity was prioritized, and the researchers’ reflective notes on the data (analytical memos) were systematically documented (Charmaz, 2006). Within this process, the relationship with the data was explicitly articulated, and the researchers’ positionality (including prior knowledge, experience, and perspectives) was incorporated into the analysis. Furthermore, in the findings section, the depth of the identified themes was demonstrated through thick descriptions and direct participant quotations, thereby enabling readers to transparently follow the analytic process (Creswell & Poth, 2016). This holistic approach aimed to enhance the study’s validity and to demonstrate that the themes were developed consistently within the research context.

## 3. Results

As a result of the data analysis, thematic analyses of the views of the child, mother and two teachers were determined separately.

### 3.1. Thematic Analysis of Data on Children

**Figure 1:**
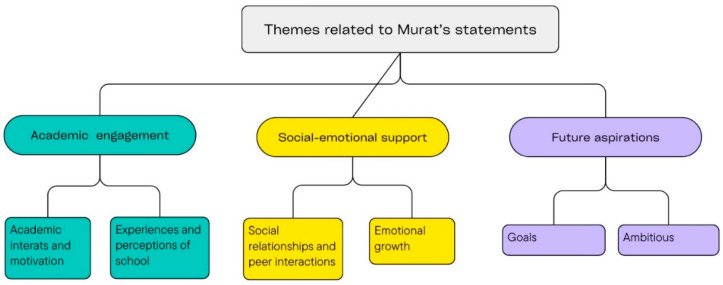
Themes related to students’ statements.

As a result of the analysis of Murat’s answers to the research questions, 3 different themes emerged in total. These themes are given below (See Figure 1).

#### 3.1.1. Academic Engagement

Murat stated that solving challenging problems in mathematics and science made him feel talented and successful. Opportunities for individual study further enhanced this sense of enjoyment. He emphasized that teachers who employed engaging and stimulating instructional methods strengthened his motivation to learn and his interest in the subjects. Murat also expressed his expectations regarding the school environment: the improvement of physical conditions and greater understanding and support from his peers. This theme reflects the multifaceted influence of the learning environment, teaching style, and social context on the student’s academic engagement.

*“My favorite subjects are mathematics and science. I enjoy these courses because solving challenging problems makes me feel more talented and successful. In mathematics in particular, I take greater pleasure when I have opportunities for individual study.”*

*The most important factor that facilitates learning is the teacher. When my teacher explains the lessons in an enjoyable way, I become more interested in the subject and feel more motivated. I am also able to concentrate better when the classroom is quiet.*

*“My expectation from school is that the physical conditions are organized and well maintained, and that classes are taught effectively in a way that makes learning easier. In addition, having understanding friends is important to me.”*

Murat’s views indicate that academic engagement is closely related not only to cognitive abilities but also to environmental conditions and the social climate. These findings align with the literature highlighting the sensitivity of twice-exceptional students with ASD to teacher attitudes, sensory sensitivities, and peer relationships (Foley-Nicpon & Assouline, 2020; Reis et al., 2022b).

#### 3.1.2. Social and Emotional Support

Murat described the social activities he preferred, such as playing games with friends at internet cafés and engaging in sports. These leisure activities were meaningful not only as enjoyable pastimes but also as opportunities for building social bonds, providing emotional relief, and supporting self-regulation. He emphasized the importance of having reliable and fun friends, noting that such relationships helped him feel more comfortable and emotionally secure. Murat’s experiences illustrate how the emotional sensitivity commonly observed in twice-exceptional students can be balanced through social support. He expressed that he valued emotional expression, but when experiencing intense emotions, he often remained silent. However, he reported finding relief when sharing his feelings with his mother. This underscores the particular importance of the parent–child relationship for emotional regulation.

Murat’s views related to this theme are presented below:

*“I most enjoy spending time with my friends at internet cafés; playing games and doing sports there is both relaxing and fun for us.”*

*“A good friend should be reliable and fun. With trustworthy friends, I can build deeper relationships and want to spend more time with them.”*

*The time I spend with my family is what makes me happiest. Especially doing activities with them brightens my day. When I am sad or angry, I usually prefer to remain silent, but sometimes I can express my feelings openly. I feel very relieved particularly, when I share these feelings with my mother.*

This theme demonstrates the selectivity of twice-exceptional students with ASD in social relationships, their difficulties in emotional expression, and the regulatory role of family bonds. In particular, the trust-based relationship established with the mother emerges as a significant factor supporting Murat’s emotional regulation.

#### 3.1.3. Future Aspirations

Murat’s future plans—aiming to attend a distinguished science high school and to study medicine at a top-tier university to become a doctor—reflect his determination and ambition. His goals reveal not only a strong academic focus but also a clear sense of purpose and direction. Murat’s success in ranking within the top 3% nationwide on the LGS exam and securing admission to a science high school further underscores his intellectual ability, dedication, and perseverance in pursuing his objectives. This theme highlights his capacity to make realistic and concrete plans for the future, driven both by personal ambition and by the desire to succeed in a demanding field. Murat’s views related to this theme are presented below:

*My future plans include attending a reputable science high school and then becoming a doctor. I aspire to find solutions that will improve the daily lives of children with disabilities. I am already working hard to achieve these goals, and every successful step brings me a little closer to my next objective.*

### 3.2. Thematic Analysis of Data Regarding the Mother

**Figure 2:**
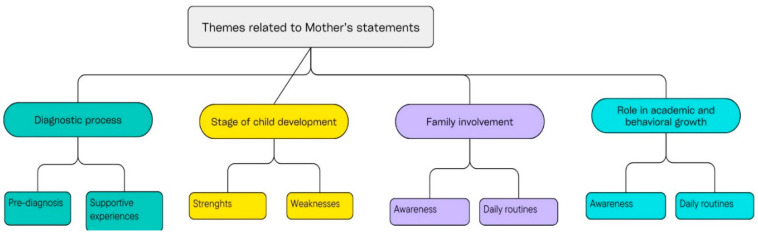
Themes related to the mother’s statements.

As a result of the analysis of the mother’s answers to the research questions, 5 different themes emerged in total. These themes are given below (See Figure 2).

#### 3.2.1. Diagnostic Process

The diagnostic process was shaped by the child’s early symptoms, such as social isolation, distractibility, and repetitive behavioral patterns. In particular, feedback from teachers and peers reinforced the family’s recognition of the need for intervention. The diagnosis was ultimately established through specialist evaluations following a referral from a pediatrician. The mother openly expressed the emotional challenges she experienced at the outset of this process and emphasized the decisive role of the support provided by teachers and institutions in helping her cope. The mother’s perspectives on this theme are presented below:

*My child constantly rocks, wants to talk about his own areas of interest, and generally prefers to play alone. He also becomes very distressed when his routines are disrupted. His teachers reported that he was distractible, and he was excluded by his peers.*

*When my child was eight years old, during a visit to a pediatric specialist for another health concern, this condition was noticed. We conducted further research, consulted with specialists, and eventually received the diagnosis. Although it was frightening at first, our findings showed that his condition was mild and accompanied by giftedness, which made things easier to manage. After the diagnosis, both the teachers and the institutions were very supportive in helping me cope with the challenges I faced throughout the process.*

This theme highlights that the diagnostic process in 2e individuals unfolds as a multilayered experience, both academically and emotionally. The mother’s emotional responses to the diagnosis and her reliance on support mechanisms align with findings in the literature that emphasize the importance of post-diagnostic family adaptation and school–family collaboration (Assouline et al., 2009). In this respect, the diagnostic process can be viewed as a critical threshold that necessitates the reorganization of the entire system alongside the child.

#### 3.2.2. Stages of Child Development

This theme examines the interaction between the child’s evolving strengths and developmental challenges across preschool, primary, and middle school years. In preschool, the child struggled with establishing eye contact and engaging in peer interactions, yet demonstrated remarkable strengths such as quickly grasping and retaining new concepts. During primary school, his capacity for rapid learning and knowledge acquisition became more pronounced, while difficulties in forming social relationships persisted. By middle school, his development reflected progress in self-regulation and self-control, indicating greater maturity. However, his emotional sensitivity and intensity continued, underscoring the need for support not only in academic and behavioral domains but also in emotional regulation and psychological resilience.

The mother’s perspectives on this theme are presented below:

*During preschool, my child experienced difficulties with socialization because he was unable to make eye contact and constantly wanted to talk about his own interests. However, his ability to quickly grasp everything that was taught to him was a notable strength. In primary school, he learned and absorbed knowledge rapidly, taking it to the next level, yet he did not establish strong bonds with his peers. In middle school, his self-control improved, but his emotionality remained a weakness.*

#### 3.2.3. Family Involvement

This theme emphasizes the child’s specific circumstances and the family’s growing awareness of and adaptation to his unique needs. The child’s high level of activity was identified as stemming from his particular condition, which the family gradually came to understand. In response, the family proactively developed special plans to support his socialization, aiming to increase his interactions and foster a sense of belonging. The theme also addresses challenges within family dynamics and daily routines. While the child’s disciplined and structured lifestyle positively influenced family cohesion and provided stability, his hyperactivity occasionally led to disruptions. These difficulties prompted the family to seek various forms of support, such as professional guidance and community resources, to better manage the challenges. This dual emphasis on structure and external support reflects the family’s strong commitment to the child’s well-being. The mother’s perspectives on this theme are presented below:

*The fact that he is constantly on the move is due to his special situation. Since we understand this, we make plans for him to spend more time outside with his family and friends to socialize. We look for information as a family.*

*We often have difficulties because he doesn’t want to do things on his own. In order to solve these difficulties, we receive tutoring support for SACs and HSTS preparation. We also try to overcome these difficulties by determining our daily routines and providing family support.*

#### 3.2.4. Role in Academic and Behavioral Growth

In this theme, the mother provided detailed insights into her child’s academic progress, the evolving nature of his relationships with teachers, and the family’s role in addressing his emotional needs. Initially, teachers interpreted the child’s behaviors as disinterest, which led to misunderstandings of his needs. However, communication following the diagnosis resulted in a positive shift in teacher attitudes, creating a more supportive classroom environment. The mother noted that her child’s increased participation in social activities had a favorable impact on peer relationships and social skills. She also emphasized the family’s role in fostering the child’s emotional regulation, highlighting empathy and communication as decisive factors in this process.

*My child’s teachers used to think he was uninterested and unwilling because he could not make eye contact. However, after the diagnosis, their perceptions and behaviors toward him changed positively. As I explained his characteristics and maintained constant communication with the teachers, their academic support began to increase, tailored to both his strengths and weaknesses.*

*“I help my child express and regulate his emotions by showing empathy and speaking with a calm and understanding tone.”*

*Emotionally, I meet my child’s needs by hugging him often, expressing my love, going to places he wants to visit, and making plans for the things he enjoys. In addition, by participating in social activities together, I also help fulfill his emotional and social needs.*

The mother indicated that she consciously supported her child’s emotional regulation through strategies such as physical affection, expressions of love, and shared activities. This approach illustrates how the home environment functions as a regulatory context not only for academic development but also for socio-emotional growth.

### 3.3. Thematic Analysis of Data Regarding the Teachers

**Figure 3:**
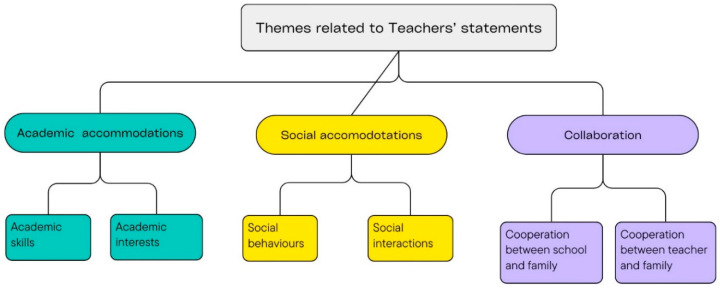
Themes related to teachers’ statements.

As a result of the analysis of the answers given by the teachers to the research questions, 3 different themes emerged in total. These themes are given below (see Figure 3).

#### 3.3.1. Academic Accommodations

This theme focuses on how the student’s strong academic abilities were addressed within the educational process and on the strategies employed by teachers to manage challenges in the classroom setting. The teachers reported that the child’s advanced perception and rapid learning skills were supported through project competitions and enriched activities. At the same time, they emphasized that his high level of activity made adherence to classroom rules difficult, posing a challenge that required management for both the student and the teachers. Although no individualized education plan specifically tailored to his interests was in place, adaptations were made by assigning research tasks aligned with his areas of curiosity. It was also noted that simple questions were used to capture his attention during the learning process and that his hyperactivity was tolerated through in-class monitoring.

*The child’s perceptual ability is very good, so I support this in project competitions. I also try to support his ability to learn quickly by giving a lot of information and providing a lot of activities with a few class hours.*

*“The child finds it very difficult to stay still; he is constantly on the move. His difficulty in obeying the classroom rules challenges both him and us.”*

*“No specific learning plan was created, but we tried to contribute to the child’s development by providing research topics according to the child’s interests. It is also supported by an enriched education program.”*

*“The child is constantly moving, you think he doesn’t understand the subject, but he listens and perceives it. During the lesson, I try to attract his attention by asking simple questions and ignoring his active nature.”*

#### 3.3.2. Social Accommodations

This theme focuses on the student’s level of social participation in the classroom, his adaptation to group activities, and the ways in which teachers supported this process. It was noted that while the student experienced difficulties in making eye contact, he was able to engage with peers at a basic level. In larger groups, he tended to remain in the background, indicating a limited yet tolerable challenge in social participation. The teachers reported that they supported his involvement in group projects to foster social skills and encouraged his active participation through shared leadership strategies. Furthermore, in cases of restlessness within the classroom, directive prompts were employed as behavioral regulation strategies.

*Although the child has difficulties with eye contact, I observe that he does not experience major problems in interacting with his peers. I cannot say that he is highly successful in social collaboration and interaction, but I also do not observe significant difficulties.*

*“The child participates in group activities to some extent. However, when the groups are large, adapting and collaborating can be challenging for him. He generally prefers to remain in the background during group activities.”*

*“To support the child’s social skill development, we encourage him to work on projects in groups with his peers. We also implement a shared leadership approach, ensuring that he takes the lead when it is his turn.”*

#### 3.3.3. Collaboration

This theme addresses the ongoing communication established between teachers and the family, and how this exchange contributes to a deeper understanding of the child’s needs. According to the interviews, the information provided by the family helped teachers to interpret the child’s classroom behaviors more accurately, which in turn fostered greater understanding and flexibility in their approaches. Teachers reported that they occasionally offered suggestions regarding the home learning process; however, they emphasized that the family’s high level of awareness and sensitivity often made additional guidance unnecessary.

*We are in constant communication with the family about the student, and we do this communication mostly via telephone. The information I receive from the family makes the child’s situation more understandable, and this gives me the opportunity to be more tolerant.*

*“I make the necessary suggestions, but frankly, his parents are very sensitive and knowledgeable about this issue. Learning at school is very good; I don’t offer any extra suggestions for the home environment.”*

## 4. Discussion

This study examines the academic and social experiences of a gifted student with ASD through the perspectives of the student himself, his mother, and his teachers. The discussion section is structured around four main themes derived from the analysis of the findings.

### 4.1. Academic Performance

This study examined the academic experiences of a gifted student with ASD through the perspectives of the student, his mother, and his teachers. The student reported feeling more competent and successful when working on challenging problems in mathematics and science, particularly when provided with opportunities for independent study. He expressed a clear preference for these subjects and emphasized the importance of interactive and engaging instructional methods in facilitating his learning process. Prior research highlights the value of instructional adaptations in fostering critical thinking, interest, and motivation among gifted learners (Kim, 2016; Rubenstein et al., 2013). As Baum et al. (2014) underscore, individualized educational approaches for gifted students with ASD align with their preference for autonomous engagement in demanding academic domains. Such approaches also address the dual demands of giftedness and ASD, thereby enabling more responsive support (Cox & Root, 2020). Moreover, Assouline et al. (2009) and Willard-Holt et al. (2013) emphasize the necessity of specialized strategies tailored to the unique needs of 2e students. These strategies are critical not only for supporting their academic achievement but also for fostering their social and emotional development.

Within the Turkish context, where educational practices for 2e students are rarely supported through individualized programs, the findings underscore the critical importance of providing opportunities for autonomous learning. Particularly in state schools, where curricular flexibility is limited, supportive strategies implemented at the teacher’s individual initiative can play a decisive role in realizing students’ potential (Yavuz & Temiz, 2025). The necessity for autonomy and context-specific academic scaffolding may be further elucidated via Bronfenbrenner’s ecological systems theory, with particular attention to the microsystem and mesosystem layers, wherein the reciprocal interactions among school, educator, and familial milieus exert a direct influence on scholastic attainments. Integrating both giftedness and ASD into educational programming creates opportunities to maximize these students’ potential, enabling them to overcome challenges and thrive in appropriately structured learning environments (Shaunessy-Dedrick & Lazarou, 2020). Tailored interventions are essential for several reasons. First, 2e students combine high abilities with difficulties such as poor literacy, low self-esteem, and organizational challenges, which necessitate differentiated educational support to address their varied needs. Second, 2e students are frequently misidentified, as their disabilities may overshadow their abilities or their giftedness may mask their challenges. Adapted interventions are thus necessary for accurate identification and effective support. Consequently, effective interventions enhance achievement, self-efficacy, and well-being by leveraging students’ strengths while addressing their difficulties through strategies such as inquiry-based learning, talent-focused environments, and extended task durations.

The findings further demonstrate that the student’s participation in project competitions and strong motivation toward scientific goals (e.g., becoming a physician), when adequately supported, significantly enhanced academic achievement. This aligns with Renzulli’s Enrichment Triad Model, which emphasizes that gifted students achieve higher levels of performance when engaged in original production and problem-solving processes (Renzulli et al., 2013). The student’s individual success exemplifies the paradox inherent in being both highly able and having a learning difficulty: on the one hand, the potential for exceptional achievement, and on the other, challenges in adhering to classroom rules. This duality requires teachers to navigate a complex pedagogical process. Accordingly, educators working with 2e students must be well-equipped in both special education and talent development domains (Bianco & Leech, 2010).

### 4.2. Social Experiences

The student, identified as gifted and diagnosed with ASD, reported a preference for spending time with friends in cafés and engaging in sports, describing these activities as both enjoyable and relaxing. He emphasized trustworthiness and playfulness as the qualities of a good friend and expressed a desire for understanding peers and a well-structured school environment. The social activities and contexts highlighted by the student underscore the vital role of social interactions for individuals with ASD. These social settings are located at the microsystem level of Bronfenbrenner’s ecological systems theory, representing the immediate social environments that significantly influence individual development. Activities such as sports and social gatherings not only provide entertainment but also create opportunities for skill development and emotional relief (Gardner et al., 2014). Social interactions further assist these students in developing social functionality and discovering values such as trust and enjoyment in friendships.

While the empirical findings do not furnish direct substantiation of the student’s engagement in social skills training initiatives, the participant’s articulated expectations pertaining to social milieus intimate a prospective necessity for such interventions, concomitant with the criticality of reinforcing supportive social architectures. In this vein, educators and school administrators could enhance the student’s academic and socio-emotional progression by instituting social skills curricula and collaborative activities aimed at nurturing reliable peer relationships. More broadly, empirical research has demonstrated the general effectiveness of such social skills interventions in supporting students’ development (Kamps et al., 1992; Koegel et al., 2013).

Research has consistently demonstrated the effectiveness of social skills training in enhancing interactions and fostering friendships among children with ASD (Bauminger, 2002; Williams White et al., 2007). Strategies aimed at cultivating these skills make significant contributions to social integration (Kasari et al., 2012). Studies conducted in Turkey have likewise shown that school-based interventions can improve social communication skills and reduce children’s perceptions of loneliness (Kabasakal & Çelik, 2010). The student further reported that family time brought him the greatest happiness and that family activities brightened his day. While he tended to remain silent when feeling sad or angry, he sometimes shared his emotions with his mother, which he found relieving. His aspiration to attend a prestigious science high school and pursue medicine reflects his strong academic motivation. These observations not only underscore the dual academic and emotional needs of twice-exceptional students with ASD but also demonstrate that family interactions provide critical emotional support and a sense of security (Hickey et al., 2019). Within this framework, the family is regarded as a primary and highly influential entity within the student’s microsystem, directly shaping emotional regulation and social interaction capabilities. This dynamic occupies a central role in Bronfenbrenner’s theoretical model. Furthermore, such experiences suggest that emotional regulation, as manifested in their behaviors, plays a key role in enhancing social interactions (Samson et al., 2015).

Several inferences can be drawn regarding the support of socio-emotional development. First, consistent parental and school support is vital for 2e students in enhancing their self-efficacy, addressing their weaknesses, and realizing their potential. Second, emphasizing strengths in education can improve self-perceptions, foster confidence, and promote positive psychological outcomes. Third, opportunities for achievement increase learning, self-esteem, motivation, and emotional well-being (Duyar et al., 2023). These implications demonstrate that, alongside individualized interventions, school climate and teacher sensitivity also shape students’ social integration. The ways in which teachers build relationships, guide peer groups, and structure social acceptance can serve as critical support mechanisms for these students (Pfeiffer, 2012). The interconnections among various settings align with the mesosystem level as delineated by Bronfenbrenner, encompassing the interrelationships among microsystems such as family, educators, and peer groups, which collectively exert a significant influence on a student’s developmental trajectory.

Following the diagnosis, educators exhibited enhanced attitudes, which appeared to facilitate the student’s learning process and improve classroom interactions. Regarding peer dynamics, the student demonstrated increased efforts toward social engagement, though no definitive evidence indicated a shift in peers’ attitudes. Academically, the educators’ heightened emphasis on the student’s strengths contributed to fostering a more supportive learning environment; however, quantifiable improvements in academic performance remained inconclusive. Williams White et al. (2007) similarly underscore the importance of social skills training in enhancing social participation among middle school students with autism spectrum disorder (ASD). Complementing this, maternal reports suggest that empathetic parental support played a crucial role in bolstering the child’s emotional regulation. Such familial involvement underscores the pivotal role of parental engagement in promoting both academic and social outcomes, as supported by Sofronoff et al. (2004).

The nature of social experiences is also shaped by individuals’ social skills and the opportunities provided by environmental structures. While some 2e students may display heightened sensitivity in social relationships, others may exhibit lower sensitivity, and such variability can contribute to relational difficulties (Foley-Nicpon, 2021). In this regard, the personalization of intervention plans for 2e students is considered essential.

### 4.3. Emotional Support

The student also expressed that spending time with family brought him the greatest happiness and that family activities brightened his day. Although he tended to remain silent when feeling sad or angry, he sometimes shared his emotions with his mother, which he found relieving. His aspiration to attend a prestigious science high school and pursue medicine reflects his strong academic motivation. In terms of family interactions and the provision of critical emotional support, the mother’s presence appeared to play a pivotal role in the child’s emotional and social engagement. In this context, the unconditional acceptance, empathy, and sense of security provided by the family stand out as central to the development of the student’s emotion regulation skills. This scenario exemplifies the microsystem level within Bronfenbrenner’s ecological systems theory, wherein the student’s emotional development is shaped by immediate and ongoing interactions within the family environment.

Indeed, Bagheri et al. (2017) emphasize that emotion regulation, particularly in children with exceptionalities, is shaped in conjunction with environmental sensitivities. The student’s mother closely observed his tendencies to suppress or express emotions and encouraged him to articulate them when necessary. Such parental responses enhance children’s capacity to label, express, and regulate emotions (Denham et al., 2007). Moreover, the student’s tendency to set ambitious goals and develop long-term plans constitutes important indicators of emotional resilience and self-efficacy (Bandura, 1997). Gifted students, in particular, often demonstrate future-oriented aspirations and a strong achievement motivation (Subotnik & Rickoff, 2010). Aspirations such as pursuing advanced degrees in science reflect both their high levels of motivation and their capacity for long-term planning. Reis and Renzulli (2023) highlight the impact of such goals on academic motivation and the importance of supportive structures in fostering achievement. The relationship between future orientation and emotional support in such students is bidirectional; while the desire to attain one’s goals amplifies the need for emotional support, the support received simultaneously strengthens the potential to achieve those goals (Rinn & Majority, 2018). However, emotional support should not be limited to the family context. Teachers’ provision of an emotionally secure environment also positively influences students’ attitudes toward learning. The student’s trust-based relationship with his teacher reduced his hesitation to express himself and enhanced his classroom participation. In this regard, emotional support functions not only as a facilitator in coping with emotions but also as a determinant of the quality of academic interactions. Furthermore, the combined impact of interactions between family and teachers on the student aligns with Bronfenbrenner’s mesosystem concept, as the interdependent relationship between these two microsystems fosters the student’s social and emotional well-being.

### 4.4. Teacher–Family Collaboration

Teachers reported maintaining continuous communication with the family to ensure coordination between school and home environments, noting that this interaction was conducted primarily via telephone. Such communication facilitated a deeper understanding of the student’s condition and fostered a more tolerant approach on the part of the teachers. Although no additional learning recommendations were provided, the family was perceived as knowledgeable and attentive. This type of collaboration is critical for twice-exceptional students with ASD, as it enhances both their academic and social outcomes (Josilowski & Morris, 2019). Regular communication enables tailored interventions and allows for a more nuanced understanding of the student’s challenges (Epstein, 2002). The family’s proactive involvement further illustrates that they were well equipped to support the student’s needs (Christenson & Sheridan, 2001). These reciprocal interactions align with the mesosystem level of Bronfenbrenner’s ecological systems theory, which delineates the framework through which interconnections among various microsystems, such as family and school, shape individual development.

These findings hold particular significance for educational contexts in Türkiye. Since there is no specific policy framework addressing the needs of twice-exceptional students, teacher–family collaboration often relies on individual efforts. In this regard, systematic and sustainable communication between teachers and families plays a balancing role in addressing the multilayered needs of 2e students. Notably, teachers’ ability to integrate family-provided observations—such as behavioral patterns at home and preferred modes of learning—into classroom practices supports both academic achievement and socio-emotional development. This interactive process exemplifies the mesosystem level within Bronfenbrenner’s ecological systems theory, wherein individual development is influenced by the interconnections forged between distinct environmental contexts, such as the home and school settings. Sheridan et al. (2019) emphasize that interventions grounded in teacher–family partnerships enhance students’ school performance, foster mutual confidence between families and teachers, and strengthen students’ sense of self-efficacy. These findings underscore the necessity of contextually responsive, individualized, and empowering support strategies in the education of twice-exceptional students. These requirements correspond closely with Bronfenbrenner’s ecological systems framework, which underscores the interconnectedness of environmental contexts and promotes a multilayered perspective on developmental interactions.

## 5. Conclusions

This study highlights the challenges faced by a gifted student with ASD within a unique context and underscores the need for tailored strategies to better understand and address such cases. Within this specific case, the student demonstrated greater achievement and motivation in mathematics and science when provided opportunities for independent study, while a quiet environment and effective teacher interaction further enhanced his performance. Although the findings are not intended to be generalized, they offer insights into potential strategies for students with similar profiles. Considering this case, individualized educational strategies and appropriately structured learning environments appear to be effective in supporting the realization of student potential. Enriched programs and participation in project-based competitions further fostered academic success by capitalizing on the student’s rapid learning capacity. From a social perspective, activities such as spending time in internet cafés and engaging in sports contributed to the development of social skills, although adapting to larger peer groups remained a challenge. Teacher encouragement through shared leadership and group work promoted greater social adjustment. Finally, the strong emotional bond with the mother and the overall family support were crucial in fostering both academic and socio-emotional development. In this regard, the findings underscore the pivotal role of family–teacher collaboration in addressing the complex needs of twice-exceptional students.

The limitations of this study stem primarily from its case study design and the small sample size. The research involved a single student diagnosed with ASD and identified as gifted, along with his mother and two teachers. Consequently, the findings cannot be generalized to a broader population. Nevertheless, these limitations simultaneously constitute the contextual strength of the study by offering an in-depth understanding of interactions within a unique educational and social environment. The detailed account of this individual case provides specific insights that may inform practices in comparable contexts. Furthermore, the exclusive reliance on qualitative data constrains the objectivity of the results. Since the study was conducted solely within the Turkish context, its findings may not be directly transferable to educational systems in other countries. Future research should expand the sample size, include more diverse demographic characteristics, and examine different age groups, socio-economic backgrounds, school types, and teacher training practices. In addition, strategies for enhancing teacher awareness and support mechanisms for families of twice-exceptional students should be incorporated into future research agendas.

## Figures and Tables

**Table 1:** Demographic Profile of Study Participants.

Participant	Age	Educational Level/Field	Institutional Affiliation	Gender	Teaching Experience
Murat (Student)	8th grade (13 years)	Middle School	State school and SACs	Male	-
Mother	45	High School Graduate	Housewife	Female	-
Teacher	33	Science and Technology	SACs	Female	3 years
Teacher	35	Mathematics Education	SACs	Female	5 years

## Data Availability

The data that support the findings of this study are available on request from the corresponding author. The data are not publicly available due to privacy or ethical restrictions.

## Abbreviations

The following abbreviations are used in this manuscript:

ADHD	Attention Deficit Hyperactivity Disorder
ASD	Autism Spectrum Disorder
ASIS	Anatolian-Sak Intelligence Scale
HSTS	High School Transition System
LD	Learning Disability
SAC	Science and Art Center
2e	Twice-exceptional
